# Management of chylous ascites following pancreaticobiliary surgery

**DOI:** 10.1002/jgh3.12179

**Published:** 2019-04-24

**Authors:** Harjeet Singh, Narendra Pandit, Gautham Krishnamurthy, Rajesh Gupta, Ganga R Verma, Rajinder Singh

**Affiliations:** ^1^ Department of General Surgery Post Graduate Institute of Medical Education and Research (PGIMER) Chandigarh India

**Keywords:** chyle leak, pancreaticoduodenectomy, surgery

## Abstract

**Background:**

Chyle leak is an uncommon form of ascites occurring due to the accumulation of lipid‐rich lymph into the peritoneal cavity. Traumatic injury to the lymphatic system due to pancreaticobiliary surgery can lead to this phenomenon.

**Method:**

We retrospectively evaluated the data of 159 patients of pancreticobiliary surgery from January 2012 to December 2016. Five patients (5/137, 3.6%) sustained a chylous leak following pancreaticoduodenectomy and one patient (1/22, 4.5%) sustained a chylous leak following Roux‐en‐Y hepaticojejunostomy for postcholecystectomy biliary stricture.

**Results:**

Average daily output was 441 mL (*range*: 150–800 mL/day), and total duration of output was 16.5 days (*range*: 4–35 days). Mean hospital stay increased to 19.1 days (*range*: 10–40 days). All the patients were successfully managed conservatively with a combination of customized enteral feeds, supplemental parenteral nutrition, and octreotide. One patient required additional percutaneous drainage.

**Conclusion:**

Chyle leak can be successfully treated with conservative management but at the cost of increased hospital stay.

## Introduction

Chylous ascites, first described by Morton in 1694, is an uncommon form of ascites characterized by a creamy‐milky fluid rich in triglycerides.^1^ A chyle leak occurs frequently after aortic surgery and retroperitoneal lymph node dissection in urological surgery^2^, ^3^ but can also complicate pancreatobiliary surgery in 1.1–16.3% of cases.^4^, ^5^ Disruption of visceral lymphatic channels, including cisterna chyli during dissection, leads to chyle leak.^6^

Chylous fluid is rich in triglycerides, lymphocytes, and immunoglobulin; hence, a large‐volume loss leads to increased morbidity because of metabolic consequences, that is, malnutrition, immunodeficiency, and even infections.^7^ The occurrence of this complication after hepaticojejunostomy in benign biliary stricture (BBS) has not been described in English literature. This paper highlights the possible anatomical site and route of lymphatic channel disruption during pancreaticoduodenectomy and hepaticojejunostomy (Fig. 2). This report also re‐emphasizes the importance of conservative management for this complication in pancreatic resections and hepaticojejunostomy and revisits the management of chylous ascitis.

## Methods

A retrospective review of a prospectively maintained database of patients who developed chylous leak/ascites following pancreatic resections for malignancy or chronic pancreatitis and hepaticojejunostomy for BBS between July 2012 and March 2015 were analyzed. The records were reviewed, paying attention to type of surgery, timing of appearance of chylous ascites, and outcome of management. Chylous ascites was defined as ≥100 mL/day of milky drain output with triglyceride contents ≥ 110 mg/dL. It should be culture negative and free from bile and amylase.

## Results

**Figure 1:**
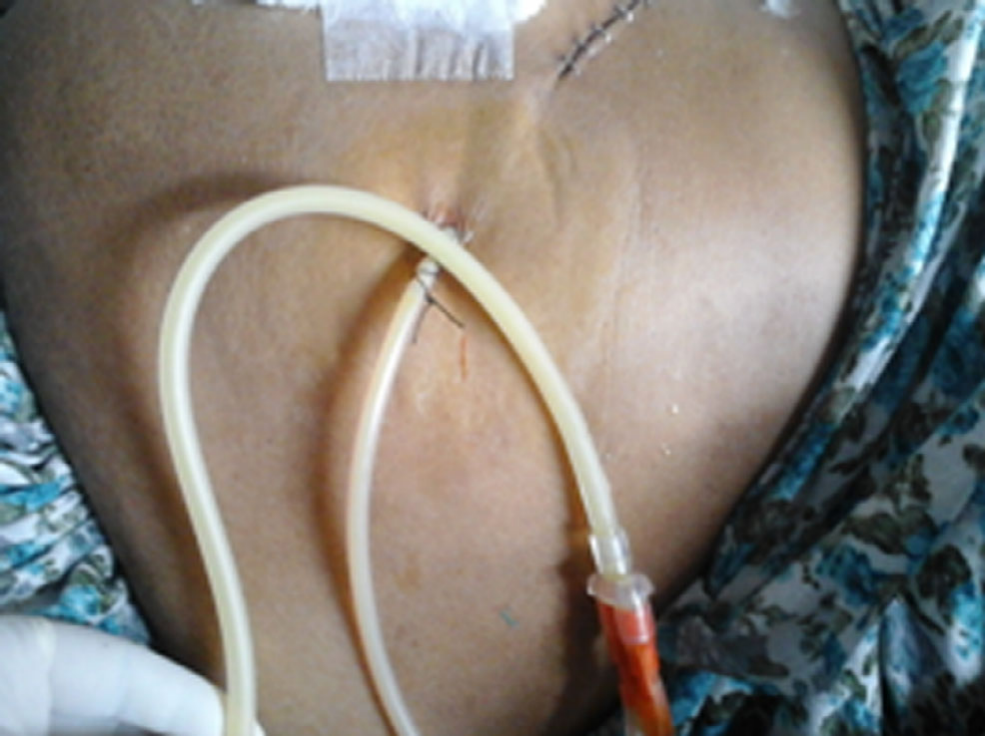
Intraoperatively placed left peripancreatic closed suction drain following pancreaticoduodenectomy to drain chyle.

A total of 137 pancreaticoduodenectomies and 22 hepaticojejunostomies in BBS were performed. The overall incidence of chylous leak/ascites was 3.77% (6/159). Five patients developed this complication following pancreaticoduodenectomy (3.64%) and one patient following Roux‐en‐Y hepaticojejunostomy. Four of five pancreaticoduodenectomies were performed for periampullary carcinoma, and in one patient, the indication was groove pancreatitis. None of the patients received neoadjuvant therapy. The demographics of patients are given in Table 1. The median age for the six patients was 40 years (*range*: 25–65), with a male:female ratio of 1:1. Feeding jejunostomy was performed in all patients, and milk‐based feed rich in protein and fat was administered after 48 h of surgery. Three patients developed chylous ascites (with diffuse peritoneal fluid), while the other three had chylous leak through the drain (Fig. 1) The mean time to the detection of chyle leak was 5 days after surgery, coinciding with the initiation of full‐strength milk‐based enteral feed. However, the earliest leak was detected on postoperative day 3.

**Table 1:** Patients' demographics and outcome

Factor	Patient 1	Patient 2	Patient 3	Patient 4	Patient 5	Patient 6
Age (years)	65	25	40	55	45	37
Gender	Female	Female	Male	Male	Female	Male
Diagnosis	Periampullary carcinoma	Biliary stricture (postcholecystectomy)	Periampullary carcinoma	Periampullary carcinoma	Periampullary carcinoma	Groove pancreatitis
Surgery	Pancreaticoduodenectomy	Roux‐en‐Y Hepaticojejunostomy	Pancreaticoduodenectomy	Pancreaticoduodenectomy	Pancreaticoduodenectomy	Pancreaticoduodenectomy
Average output (mL/day)	250	800	700	250	150	500
Chylous leak/Chylous ascites	Chylous leak	Chylous ascites	Chylous ascites	Chylous leak	Chylous leak	Chylous ascites
Duration of drainage (days)	7	35	28	20	4	5
PPN	No	Yes	Yes	No	No	No
Octreotide	No	Yes	Yes	No	No	No
Hospital stay (days)	10	40	15	30	10	10
Mortality	No	No	No	No	No	No
Follow up	Alive at 14 months	Alive at 28 months	Alive at 23 months	Alive at 20 months	Alive at 8 months	Alive at 6 months

The mean volume and duration of chyle was 441 mL/day (150–800 mL) and 16 days (4–35 days), respectively. The mean hospital stay of these patients was 19.1 days (10–40 days) (Table 1). Conservative treatment comprised customized enteral feed of high‐protein, low‐fat diet (rich in medium‐chain triglycerides) and supplemental parenteral nutrition. Octreotide of 100 μg thrice daily for 10 days was given to two patients demonstrating high chyle output, >700 mL/day. One of them also required temporary per cutaneous ascitic fluid drainage. Three patients of chyle leak without ascites responded well to conservative management alone and had earlier resolution. None of them had associated pancreaticojejunostomy or hepaticojejunostomy leak. All patients are alive and well at median follow up of 17 months (6–28 months) (Table 1).

## Discussion

The incidence of a chyle leak after pancreaticoduodenectomy is 1.8–11.0%.^8^, ^9^ Kocherization of the duodenum up to the aorta is a standard step during pancreaticoduodenectomy to facilitate resection and to remove the lymph nodes for R0 resection. Chyle leak is a result of inadvertent injury of lymphatic vessels or cysterna chyli that lie near the origin of superior mesenteric artery, above and on the posterior surface of the pancreas. Aalami et al.^7^ hypothesized that the causes of chyle ascites could be either the obstruction of the lymphatic channels leading to dilatation and/or traumatic injury to the lymphatic channels. Besides the extirpation of lymphatic vessels, even the excision of lymph nodes leaves a number of lymph vessels open in the retro pancreatic region. Pan et al.^10^ have reported that manipulation in the paraaortic area and around the root of superior mesenteric artery significantly increases the incidence of chylous ascites. Although the radical lymph node resection leading to high incidence of chylous ascites appears to be a plausible explanation,^11^, ^12^ a similar observation has not been reported by others.^6^, ^7^ Even though we do not perform radical or extended lymphadenectomy in pancreaticoduodenectomy, we have experienced this complication, suggesting that mere manipulation and standard lymphadenectomy can lead to the development of chyle leak due to inadvertent damage of lymphatic vessels.

**Figure 2:**
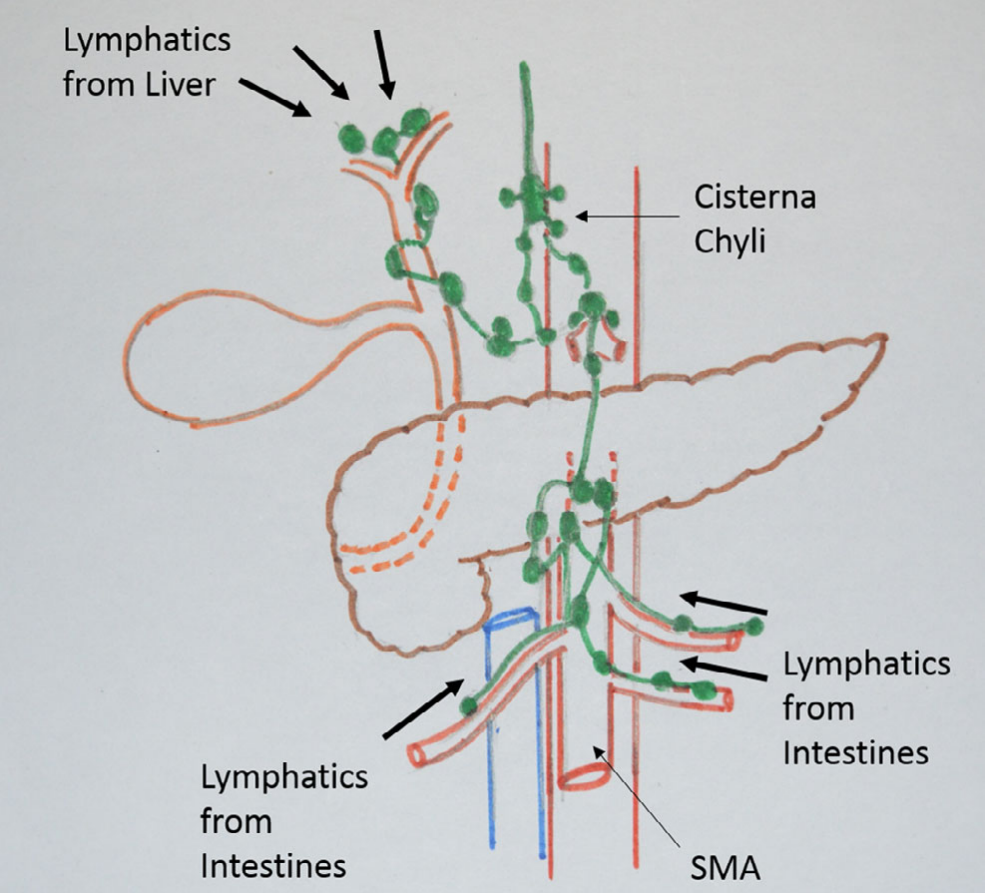
Intra‐abdominal lymphatics and lymphatic flow.

Chyle leak has been described following pancreatectomy, hepatectomy,^13^ liver transplantation,^14^ and D2 gastrectomy,^15^ but it has never been described after posthepaticojejunostomy in benign biliary disease. The porta hepatis is an important watershed of lymphatic channels. These include the large lymphatics that drain the perisinusoidal spaces in the liver and those channels that drain the extrahepatic biliary system (Fig. 2).^16^, ^17^ The localized fibrosis induced by previous injury of the common hepatic duct could have caused the obliteration of these lymphatic channels and upstream dilatation. Dissection at the porta for exposure of the ductal system could have disrupted the dilated channels, leading to chyle leak following hepaticojejunostomy. Kuboki et al.,^18^ in their experience of 17 patients of chylous ascites, reported this complication in 2 patients following combined liver and bile duct excision and attributed it to the manipulation of the hepatoduodenal ligament and resection of retroperitoneal tissue.

The chylous leak appears between 3 and 8 days postsurgery.^19^ It could be in isolation as observed by us or in association with other common postpancreatectomy complications. Strobel et al.,^20^ in their experience of 346 patients with chylous leak among 2881 patients undergoing pancreatic resection, reported an isolated chyle leak in 211 (11.1%) patients without concomitant intra‐abdominal complications and in the remaining 135 (13.8%) patients with other intra‐abdominal complications, that is, pancreatic fistula and intra‐abdominal abscess. van der Gaag et al.^21^ pointed out that the surrounding inflammation and head mass with reactive lymph nodes induced by chronic pancreatitis require more extensive dissection, leading to chyle leak. Our index patient with groove pancreatitis with bulky head of pancreas and extensive surrounding inflammation also required extensive dissection, resulting in chylous ascites. The possible reason for these chyle leaks may be extensive dissection for lymphadenectomy and use of an energy source during lymphadenectomy. The energy source may partially or temporarily seal these lymphatic channels, which can reopen and manifest as chyle leak in the early postoperative period. We suggest that, during the dissection of lymphatics in the injury‐prone area (Fig. 2), the ligation of lymphatic should be preferred over use of an energy source.

Early jejunostomy feeding following pancreaticoduodenectomy is the standard of care at our center, and chyle leak incidence of 3.7% is, in our experience, comparable to 1.3–10.8%.^5^ Some authors have suggested that total parenteral nutrition (TPN) administration from the immediate postoperative period may lower the incidence of chylous ascites.^22^ However, replacing enteral nutrition, with its proven advantages of economical cost, easy availability, being better tolerated, preserving mucosal integrity, and reducing endotoxin translocation, with TPN may not find favor amongst most clinicians. Moreover, chylous leak is a benign and easily manageable complication. It responds to conservative treatment in a large majority of cases. Abu Hilal et al.^5^ reported that, in all 40 patients, the leak stopped after MCT and supportive treatment. No patient required TPN or surgical treatment. Strobel et al.^20^ managed 346 patients with chylous ascites with or without associated intra‐abdominal complications following pancreatic resection and concluded that all patients responded to combinations of MCT, TPN, and somatostatin. In our experience as well, all patients were successfully managed with customized enteral feeding of a high‐protein, low‐fat diet administered through a feeding jejunostomy or nasogastric tube along with supportive treatment. In two of the six patients who had large‐volume chyle leak, supplementary parenteral nutrition and octreotide were used as adjuncts. Parenteral nutrition decreases the gut hormonal stimulation and decreases the chyle output. Octreotide decreases splanchnic blood flow and portal pressure, thereby reducing intestinal absorption of fat and attenuating lymph flow in major lymphatic channels.^23^, ^24^ Medium‐chain triglycerides are absorbed by enterocytes and transported as free fatty acids and glycerol directly to the liver via the portal vein.

There is no consensus for the first line of nutritional therapy following surgery.^25^ The authors feel that the continuance of enteral feed avoids the negative impact of prolonged fasting and deprivation of gut from the trophic effect of nutrition. Hence, enteral nutrition should be considered the initial mode followed by parenteral nutrition in selected cases.

To conclude, chylous leak/ascites is an uncommon complication following pancreaticobiliary surgery. It manifests within 3–7 days of surgery and can be successfully managed by customized enteral feed. Parenteral nutrition and octreotide may be required in selected cases. It prolongs hospitalization but does not lead to any major morbidity or mortality.
